# Expression of PPARα modifies fatty acid effects on insulin secretion in uncoupling protein-2 knockout mice

**DOI:** 10.1186/1743-7075-4-6

**Published:** 2007-03-06

**Authors:** Zahra Fatehi-Hassanabad, Catherine B Chan

**Affiliations:** 1 Department of Biomedical Sciences, Atlantic Veterinary College, University of Prince Edward Island, Charlottetown, PE C1A 4P3, Canada

## Abstract

**Aims/hypothesis:**

In uncoupling protein-2 (UCP2) knockout (KO) mice, protection of beta cells from fatty acid exposure is dependent upon transcriptional events mediated by peroxisome proliferator-activated receptor-α (PPARα).

**Methods:**

PPARα expression was reduced in isolated islets from UCP2KO and wild-type (WT) mice with siRNA for PPARα (siPPARα) overnight. Some islets were also cultured with oleic or palmitic acid, then glucose stimulated insulin secretion (GSIS) was measured. Expression of genes was examined by quantitative RT-PCR or immunoblotting. PPARα activation was assessed by oligonucleotide consensus sequence binding.

**Results:**

siPPARα treatment reduced PPARα protein expression in KO and WT islets by >85%. In siPPARα-treated UCP2KO islets, PA but not OA treatment significantly decreased the insulin response to 16.5 mM glucose. In WT islets, siPPARα treatment did not modify GSIS in PA and OA exposed groups. In WT islets, PA treatment significantly increased UCP2 mRNA and protein expression. Both PA and OA treatment significantly increased PPARα expression in UCP2KO and WT islets but OA treatment augmented PPARα protein expression only in UCP2KO islets (p < 0.05). PA treatment induced carnitine palmitoyltransferase I, acyl CoA oxidase and malonyl CoA decarboxylase mRNA in UCP2KO islets.

**Conclusion:**

These data show that the negative effect of saturated fatty acid on GSIS is mediated by PPARα/UCP2. Knockout of UCP2 protects beta-cells from PA exposure. However, in the absence of both UCP2 and PPARα even a short exposure (24 h) to PA significantly impairs GSIS.

## Background

In pancreatic beta cells, free fatty acids (FFA) modulate the process of glucose-stimulated insulin secretion (GSIS) [1]. Short term exposure of islets to elevated concentrations of FFA enhances GSIS whereas long term exposure results in impaired GSIS. Suppressed GSIS after FFA treatment may be caused by up-regulation in beta cells of uncoupling protein-2 (UCP2), the expression [2] and activity [3] of which is increased by FFA. UCPs (numbered 1–3 in order of their discovery) decrease metabolic efficiency by dissociating ATP synthesis from substrate oxidation in the mitochondrion [4]. The mechanism is controversial but may involve promotion of proton or fatty acid translocation [5]. In beta cells, the physiological role of UCP2 is not established. However, mild uncoupling stimulated as a consequence of variation in respiratory rate may be a fine modulator of insulin secretion [5,6]. Alternatively, UCP2 may protect islets from oxidative stress, as has been shown in brain [7]. Paradoxically, our previous study showed that feeding a high fat diet to mice lacking UCP2 enhanced beta cell responsiveness to glucose, preserved islet sensitivity to glucose and increased beta cell mass [8]. This occurred in spite of increased mitochondrial superoxide production in the beta cells of these mice [9]. Interestingly, CPTI was strongly induced in UCP2 KO mouse islets [8] and palmitic acid (PA) oxidation was enhanced leading to reduced islet triglyceride content [9]. This suggests that enzymes regulating FFA catabolism may be generally up-regulated in the absence of UCP2.

FFA may affect beta cell function subsequent to changes in the expression of enzymes related to insulin secretion via their actions on certain transcription factors. Long chain unsaturated FFA are natural, preferentially-binding ligands of peroxisome proliferator-activated receptors (PPARs) but saturated fatty acids like PA can also act as ligands [10]. The existence of both PPARα and PPARγ has been detected in pancreatic beta cells of both humans and rodents [11-13]. PPARα induces expression of UCP2 and enzymes regulating fatty acid oxidation in pancreatic beta cells [14] via PPAR response elements in target gene promoter regions [15,16]. The induction of UCP2 presumably negatively influences insulin secretion [14]. However, other studies suggest that PPARα enhances GSIS [17]. We considered that the fat oxidation phenotype displayed by UCP2 KO mice on high fat diet might be related to altered PPARα transcriptional activity. In skeletal muscle cells, intracellular generation of reactive oxygen species (ROS) leads to a down-regulation of PPARα activity [18]. ROS are elevated in UCP2 KO mice islets [9], leading to the hypothesis that UCP2 activity can modulate PPARα in islets.

To investigate this hypothesis, the UCP2 KO mouse provides a unique model. For studies of loss of function via knockdown of target gene expression, RNA interference (RNAi) has been shown to be a powerful gene suppression tool. In mammalian cells, small interfering RNA (siRNA) of 19- to 29-nt, inhibit target gene expression in a sequence-specific manner [19]. In the present study, we used a PPARα siRNA vector to suppress PPARα gene in pancreatic beta cells isolated from either UCP2KO or wild type (WT) mice in the absence or presence of saturated or monounsaturated FFA. We have also investigated the effects of these FFA on GSIS and expression of important lipogenic and lipolytic enzymes in pancreatic beta cells.

## Materials and methods

### Reagents

Collagenase (type XI), palmitic acid (PA), oleic acid (OA), BSA (fatty acid free), N-[1-(2,3-dioleoyloxy) propyl]-N,N,N-trimethylammonium methylsulfate (DOTAP) and all cell culture supplies (except as noted) were purchased from Sigma-Aldrich (Oakville, ON, Canada). TranSilent PPARα siRNA, control vectors and transBinding PPARα assay kits were purchased from Panomics (Redwood City, CA, USA). For western blotting, primary antibodies for PPARα (rabbit polyclonal IgG) and UCP2 were from ABR (Cedarlane, Burlington, ON, Canada) and Santa Cruz Biotechnology (Santa Cruz, CA, USA), respectively. β-actin primary antibody and all secondary antibodies were from Sigma-Aldrich. TRIzol, trypsin and Opti-MEM were obtained from Invitrogen (Burlington, ON, Canada).

### Animals

Female UCP2 WT or KO mice were bred from lines generated as described previously and used at 4 months of age [8]. Protocols were approved by the Animal Care Committee of the University of Prince Edward Island following the guidelines of the Canadian Council on Animal Care.

### Pancreatic islet isolation and culture

Pancreatic islets were isolated as described previously [20]. Briefly, the pancreatic duct was perfused with 3 ml of collagenase (3 mg/ml) in Hanks Balanced Salt Solution. The pancreas was then chopped into 2-mm pieces and digested by shaking (150 rpm) for a total of 40 min at 37°C. Islets were enriched by filtration [21] and then hand picked from the acinar tissue debris. Islets were cultured in Dulbecco's modified Eagle medium (DMEM) supplemented with 1% antibiotic/antimycotic, 10 mM HEPES and 8.3 mM glucose. In different wells, defatted BSA (1%), oleic acid (OA, 0.4 mM + 1% BSA) or palmitic acid (PA, 0.4 mM + 1% BSA) was added to the isolated islets for 24 h. In some experiments, PPARα knockdown with siRNA was achieved as described below.

### Islet transfection of plasmid and siRNA vectors

To optimize the tranfection protocol, islets were transfected with a plasmid containing the full length enhanced green fluorescence protein (EGFP) cDNA (1 μg/μl) using DOTAP (1:4 – 1:6 V/V of cDNA to DOTAP) following the protocol of Lakey et al [22]. Transfected islets were then incubated for 5–6 h in Opti-MEM before adding DMEM with 20% calf serum. After 24 h, the medium was replaced by fresh DMEM. After 48 h, the islets were dissociated using 0.016 % trypsin, adhered to poly-L-lysine coated glass slides, fixed in 2% glutaraldehyde solution and assayed for EGFP expression using a fluorescent microscope. The number of fluorescing cells was counted to estimate transfection efficiency under different experimental conditions. The optimal protocol (with a cDNA:DOTAP ratio of 1:6) was then used to transfect islets with PPARα siRNA or control vector (1.5 μg per transfection).

### Islet GSIS determination

Groups of 3 islets were pre-incubated in 1 ml DMEM with 2.8 mM glucose for 60 min at 37°C, 5% CO_2_. Islets were then incubated for 90 min at the indicated glucose concentration (2.8, 5.5, 11, 16.5 or 22 mM glucose) to assess insulin secretion. At the end of a GSIS experiment, the supernatant was decanted to a fresh tube and insulin was solubilized from the pelleted islets by adding 3% acetic acid. All samples were frozen at -20°C until insulin was quantified by radioimmunoassay.

### Western blotting

For PPARα (140 μg of total islet protein) and UCP2 (100 μg of total islet protein) protein expression, pancreatic islets were lysed with Nonidet P-40 (0.5%) added to the protein extraction buffer containing protease inhibitor. Total protein was separated by 12% SDS-PAGE with a 5% stacking gel. Then, the gel was electrotransferred onto nitrocellulose membranes (Trans-Blot, Bio-Rad, Toronto, Canada) and blocked with 5% skim milk for 1 h. The membrane was incubated in primary antibody (anti-PPARα, 1:500 or anti-UCP2, 1:2500), diluted in 0.1% Tween Tris-buffered saline (TTBS) overnight at 4°C. Subsequently, the membrane was incubated in secondary antibody (PPARα: anti-rabbit IgG HRP conjugate, 1:1000; UCP2: rabbit anti-goat HRP conjugate, 1:10 000) diluted in 0.1 % TTBS, for 2 h at room temperature. Specific signals were detected using enhanced ECL Plus reagent (GE Healthcare, Mississauga, ON, Canada). Protein loading was normalized using a house keeping gene antibody (mouse anti β-actin, 1:10 000 and anti-mouse IgG HRP, 1:5000).

### RNA isolation and cDNA synthesis

Total RNA was extracted from 40 isolated islets from WT or UCP2KO mice by TRIzol isolation technique. According to the manufacturer's instructions, cDNA was synthesized from 1 μg total RNA using a Cloned AMV First-Strand cDNA Synthesis kit (Invitrogen).

### Real time PCR

Real time PCR reactions were carried out using 1 μg of cDNA. PCR products were quantified fluorometrically using SYBR Green (Bio-Rad, Toronto, ON, Canada) in a Rotor Gene RG-3000 (Corbett Life Sciences, Montreal, PQ, Canada). Glyceraldehyde-3-phosphate dehyrogenase (GAPDH) expression in each sample was used as a control. Table 1 shows the sequences of the primers (forward and reverse) used for amplification. PCR amplification was performed for 40 cycles with 10 s at 95°C, 15 s at 60°C and 20 s at 72°C. Data were expressed as fold increase in the specific gene expression as compared with GAPDH expression.

**Table 1:** Sets of primers for real-time PCR analysis

Primer Name	Sequence
PPARα	forward: 5'GGGCTCTCCCACATCCTT3' reverse: 5'CCCATTTCGGTAGCAGGTAGTC3'
UCP2 [8]	forward: 5'CAGCCAGCGCCCAGTACC3' reverse: 5'CAATGCGGACGGAGGCAAAGC3'
GAPDH [8]	forward: 5'GTGGCAGTGATGGCATGGAC3' reverse: 5'CAGCACCAGTGGATGCAGGG3'
ACO [53]	forward: 5'ATATTTACGTCACGTTTACCCCGG3' reverse: 5'GGCAGGTCATTCAAGTACGACAC3'
CPT1 [50]	forward: 5'TTCACTGTGACCCCAGACGG3' reverse: 5'AATGGACCAGCCCCATGGAGA3'
MCD [54]	forward: 5'TGTTCTGATGGGCCAGGCTTACTT3' reverse: 5'TAGAGCTTTCTGAAGGCACAGGCT3'
ACC [55]	forward: 5'TGGATCCGCTTACAGAGAGACTTTT3' reverse: 5'GCCGGAGCATCTCATTCG3'

### Nuclear protein extraction and PPARα activity assay

Isolated islets from UCP2KO and WT mice were cultured as described above. Nuclear extracts were prepared according to the kit instructions and kept at -80°C until analysis. Activated PPARα from nuclear extract was measured by its DNA binding to immobilized oligonucleotide containing a PPAR consensus binding site using the TransBinding PPARα assay kit. Binding was assessed by measured absorbance at 450 nm.

### Fatty acid oxidation

Oxidation of PA was measured in islets from UCP2KO and WT mice as described [9].

### Statistical analysis

All data are expressed as means ± SE. Statistical significance was assessed by using either two-way ANOVA followed by Tukey-Kramer test or unpaired Student t test. A *P *value of less than 0.05 was considered statistically significant.

## Results

### Transfecting plasmid DNA with DOTAP in mouse islets

**Figure 1:**
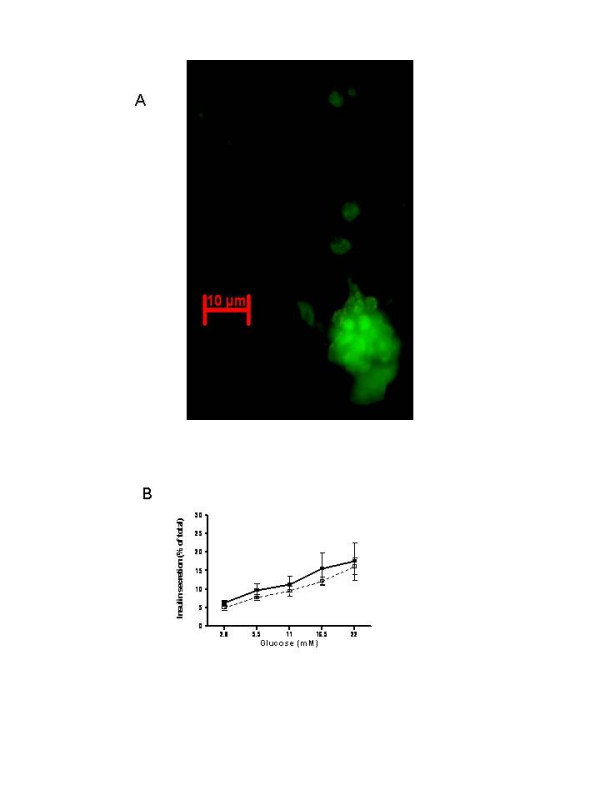
Optimization of islet transfection protocol. **(A) **Intact islets transfected with DOTAP at a DNA/liposome ratio of 1:6 (V/V). Transfected cells within the islets appear green. **(B) **Glucose stimulated insulin release in control (open squares, n = 6) and DOTAP-treated islets (closed squares, n = 4). Insulin secretion (as % of total islet content) was measured in response to different concentrations of glucose. DOTAP did not modify insulin secretion.

To optimize islet tranfection, the EGFP cDNA:DOTAP ratio was varied from 1:4 to 1:6 and EGFP expression examined by fluorescence microscopy. At a DNA/DOTAP ratio of 1:6 (V/V), DOTAP effectively allowed transfection of pancreatic islet cells with an EGFP-encoding plasmid (Figure 1A). When measured by assessing fluorescence of individual beta cells from trypsin-dispersed islets, the transfection efficiency was 85 ± 2 % (n = 8). EGFP transfection did not change insulin secretion in response to different glucose concentrations compared to untransfected islets (Figure 1B). PPARα protein expression was not modified by DOTAP treatment of the islets (data not shown).

### PPARα knock-down potentiates GSIS

**Figure 2:**
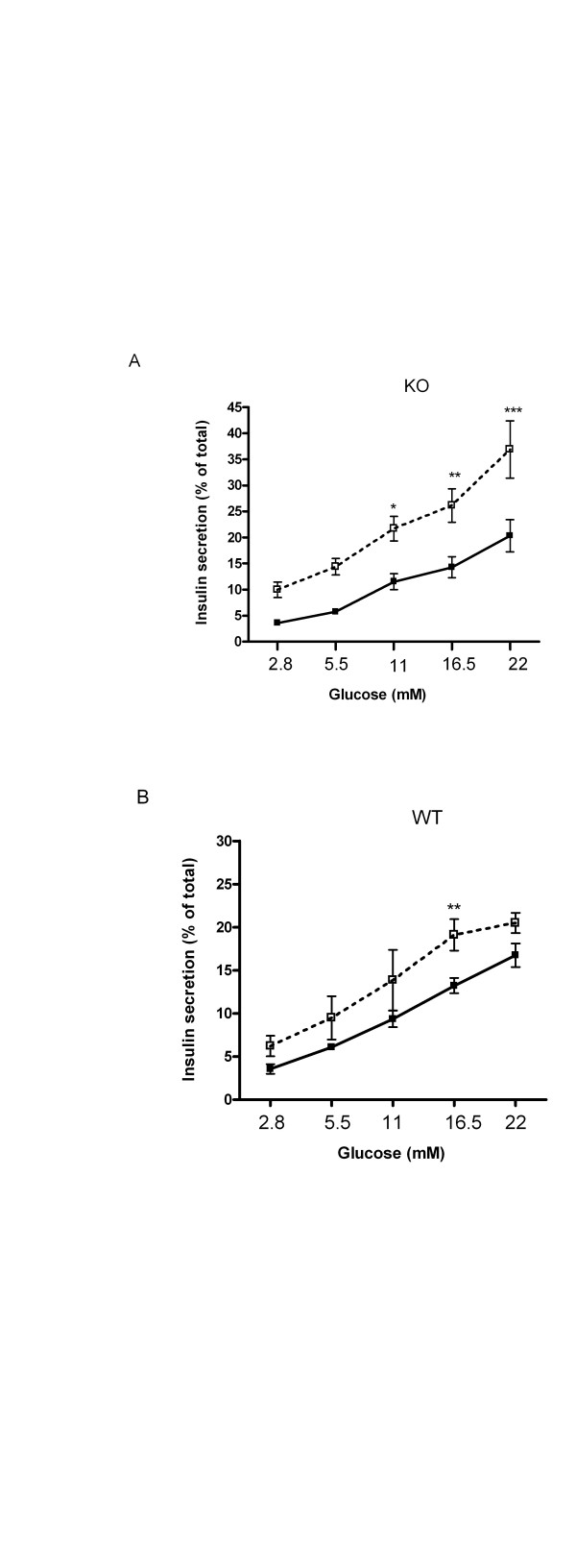
Glucose stimulated insulin release in UCP2 KO **(A) **and WT **(B) **islets. In UCP2 KO and WT islets, insulin secretion was measured in response to different concentrations of glucose in islets transfected with siPPARα (open squares, n = 5–6) or siC (closed squares, n = 8–9). *p < 0.05, **p < 0.01, ***p < 0.001 comparing response to glucose in siPPARα vs siC islets.

In a first series of experiments, we compared the effects of siPPARα with those of control vector (siC) on the function of UCP2 KO and WT isolated pancreatic islets. Knockdown of PPARα was >85% in both groups (Table 2, see also below). With increasing glucose concentrations GSIS was increased in siC treated UCP2KO and WT islets (Figure 2A &2B). In UCP2KO siPPARα-treated islets, the stimulatory effect of glucose on insulin secretion was significantly potentiated by ~75%, particularly at glucose concentrations ≥11 mM (Figure 2A, effect of glucose; P < 0.001; effect of siPPARα; P < 0.001). siPPARα treatment had a less marked effect (~50%) on insulin secretion from WT isolated islets at 16.5 mM glucose (Figure 2B, effect of glucose; P < 0.01, effect of siPPARα; P < 0.01).

**Table 2:** Effect of transilent siRNA PPARα transfection (1.5 μg, overnight) on PPARα expression (relative to β-actin) in islets isolated from UCP2 KO mice and WT mice (compared to the transilent siRNA control vector). Data are expressed as means ± sem (n = 4–7).

Groups	Control vector	Transilent PPARα vector
UCP2 KO	0.349 ± 0.136	0.029 ± 0.02
WT	0.242 ± 0.116	0.024 ± 0.007

### Effects of PA and OA on GSIS after siPPARα knockdown

**Figure 3:**
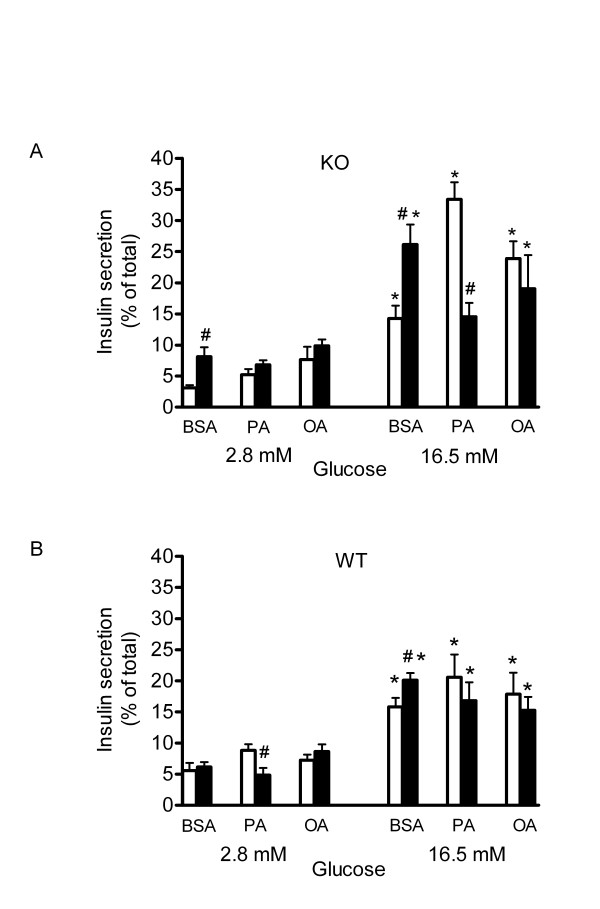
Effect of siPPARα and FFA on glucose-stimulated insulin secretion. Glucose-stimulated insulin release by UCP2 KO **(A) **and WT islets **(B) **after treatment with either control medium (defatted BSA, 1%), palmitic acid (PA, 0.4 mM + BSA) or oleic acid (OA, 0.4 mM + BSA) for 24 h in the presence of transilent siRNA control vector (open bars, n = 6) or transilent siPPARα in UCP2 KO islets (closed bars, n = 6). Two-way ANOVA performed on insulin release in response to 2.8 or 16.5 mM glucose showed a significant difference among treatment groups (P < 0.0001). Results of Tukey-Kramer multiple comparison test at each treatment showed a significant difference between siC and si PPARα (*P < 0.05 for effect of 16.5 mM glucose compared with matched 2.8 mM glucose groups; # P < 0.01 for effect of si PPARα).

To examine the effects of FFA on GSIS in the absence or presence of PPARα knockdown, WT or UCP2KO islets were incubated 24 h following siRNA transfection with PA (0.4 mM + 1% BSA) or OA (0.4 mM + 1% BSA). Results for UCP2KO islets are shown in Figure 3A. In UCP2 KO siC-treated islets, PA and OA did not modify basal insulin secretion in response to 2.8 mM glucose but increasing the glucose concentration to 16.5 mM significantly augmented insulin secretion by 4-6-fold in BSA, PA and OA treated groups. Treatment of UCP2KO islets with siPPARα significantly increased basal insulin secretion by 2-fold in the BSA treatment group. In siPPARα treated UCP2KO islets, 16.5 mM glucose significantly stimulated insulin secretion by 2-fold in both FFA treatments. However, after PPARα knockdown the response to 16.5 mM glucose of UCP2KO islets was significantly reduced ~50% in the PA-incubated group compared with siC whereas the response to OA was not altered by PPARα knockdown (Figure 3A).

In siC-WT islets (Figure 3B), FFA did not modify basal or glucose-stimulated insulin release. Likewise, neither PA nor OA affected basal insulin secretion in siPPARα-WT islets. However, in siPPARα-compared to siC-treated WT islets, insulin secretion in response to 2.8 mM glucose was significantly decreased by PA. After PPARα knockdown, and compared to 16.5 mM glucose in siC groups, insulin release was significantly increased by 25% in the BSA-treated group. PPARα expression levels had no effect on GSIS in PA- or OA-cultured WT islets.

### Induction of UCP2 by PA

**Figure 4:**
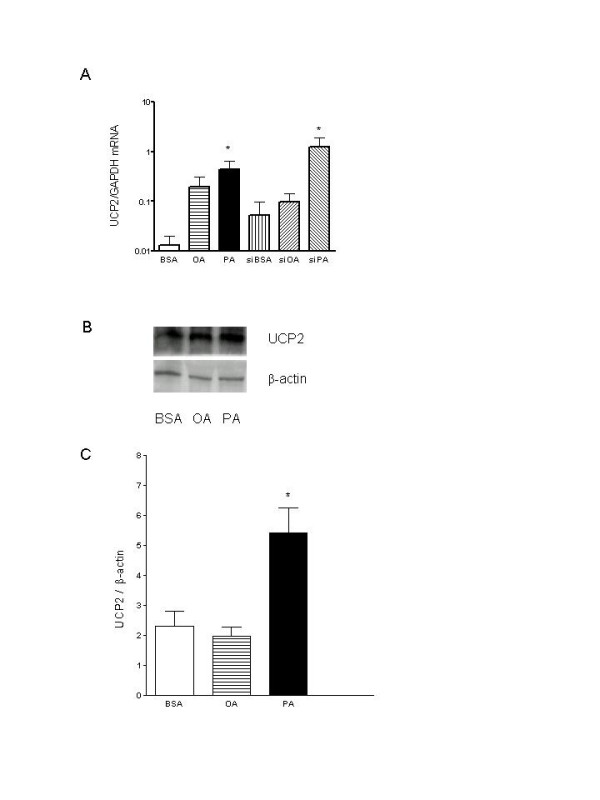
The expression of UCP2 mRNA (**A**) and UCP2 protein expression (**B **and **C**) in WT islets. UCP2 expression was determined by quantitative RT-PCR in WT islets after 24 h incubation with either BSA (open bars), OA (horizontal bars) or PA (closed bars) and also in those pre-treated with siPPARα then cultured with BSA (vertical bars), OA (left-hatched bars) and PA (right-hatched bars). The same treatment and incubation time was applied to detect the UCP2 protein expression by Western blotting. Treatment of WT islets with PA for 24 h significantly increased UCP2 mRNA and protein expression (*P < 0.05 vs BSA or siBSA). Data are presented as means ± SE, N = 5 or greater for each group.

No UCP2 mRNA or protein was detected in UCP2 KO mouse islets either in BSA- or PA-treated groups (data not shown). Culture of WT islets in 0.4 mM PA for 24 h caused a significant increase in UCP2 mRNA (Figure 4A). A similar but non-significant trend was seen for OA. Culture of WT islets with siC did not modify UCP2 mRNA expression (not shown). Culture of WT islets pretreated with siPPARα either in BSA or OA caused a non-significant increase in UCP2 mRNA expression (Figure 4A). However, there was a significant increase in UCP2 mRNA in siPPARα-treated WT islets cultured with 0.4 mM PA for 24 h (Figure 4A). UCP2 protein expression was also significantly increased in PA- but not OA-treated WT islets (Figure 4B and 4C).

### Effect of UCP2 on PPARα mRNA and protein expression in mouse pancreatic islets

**Figure 5:**
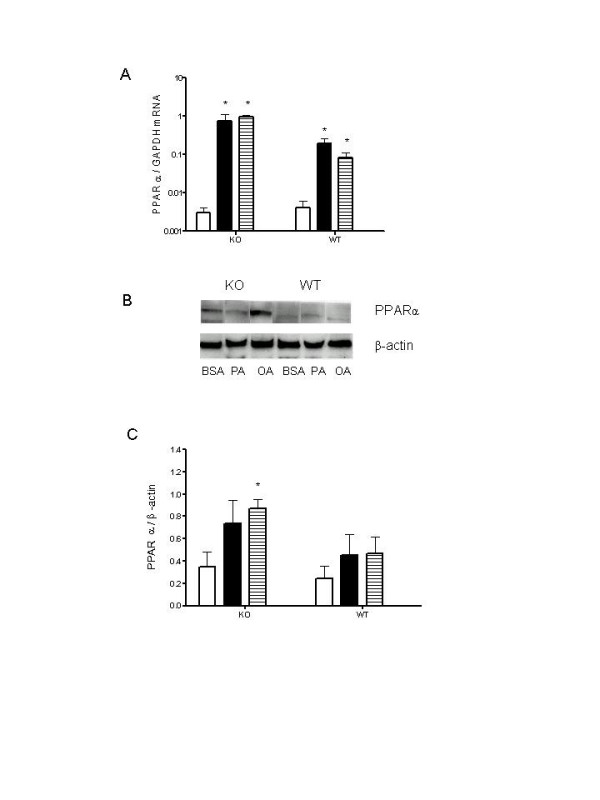
The effects of BSA, PA or OA on PPARα mRNA (**A**) and protein expression (**B**). Groups of islets isolated from KO and/or WT mice were exposed to control media (BSA, open bars), PA (closed bars) or OA (dashed bars) for 24 h. Treatment of WT and KO islets with PA and OA significantly increased PPARα mRNA expression compared to BSA (*P < 0.05). PPARα protein expression was significantly increased only in KO islets treated with OA (*P < 0.05). Two-way ANOVA performed on PPARα protein expression in KO and WT islets showed a significant difference between two genotypes (P < 0.05). Data are presented as means ± SE, N = 4–6 for each group.

To assess the effect of UCP2KO on PPARα mRNA expression, we performed real-time PCR. PPARα gene expression was not significantly different between UCP2KO and WT islets (Figure 5A). Incubation of WT and UCP2KO islets with PA and OA caused a significant induction (~100-fold) in PPARα mRNA (Figure 5A).

Similar PPARα protein expression was detected in control islets isolated from both UCP2KO and WT mice (Figure 5B). Treatment of UCP2KO islets with OA caused a 3-fold increase in PPARα expression (Figure 5B) but the effect of PA (~2-fold) was not statistically significant (Figure 5B). In WT islets, treatment with PA and OA did not change PPARα protein expression (Figure 5B). However, transfection of isolated islets from UCP2KO mice and WT mice with siPPARα caused a marked reduction in the amount of PPARα protein expression (87% and 90% in UCP2 KO and WT islets, respectively) compared to siC (Table 2).

### PPARα activity in WT and UCP2KO islets

**Figure 6:**
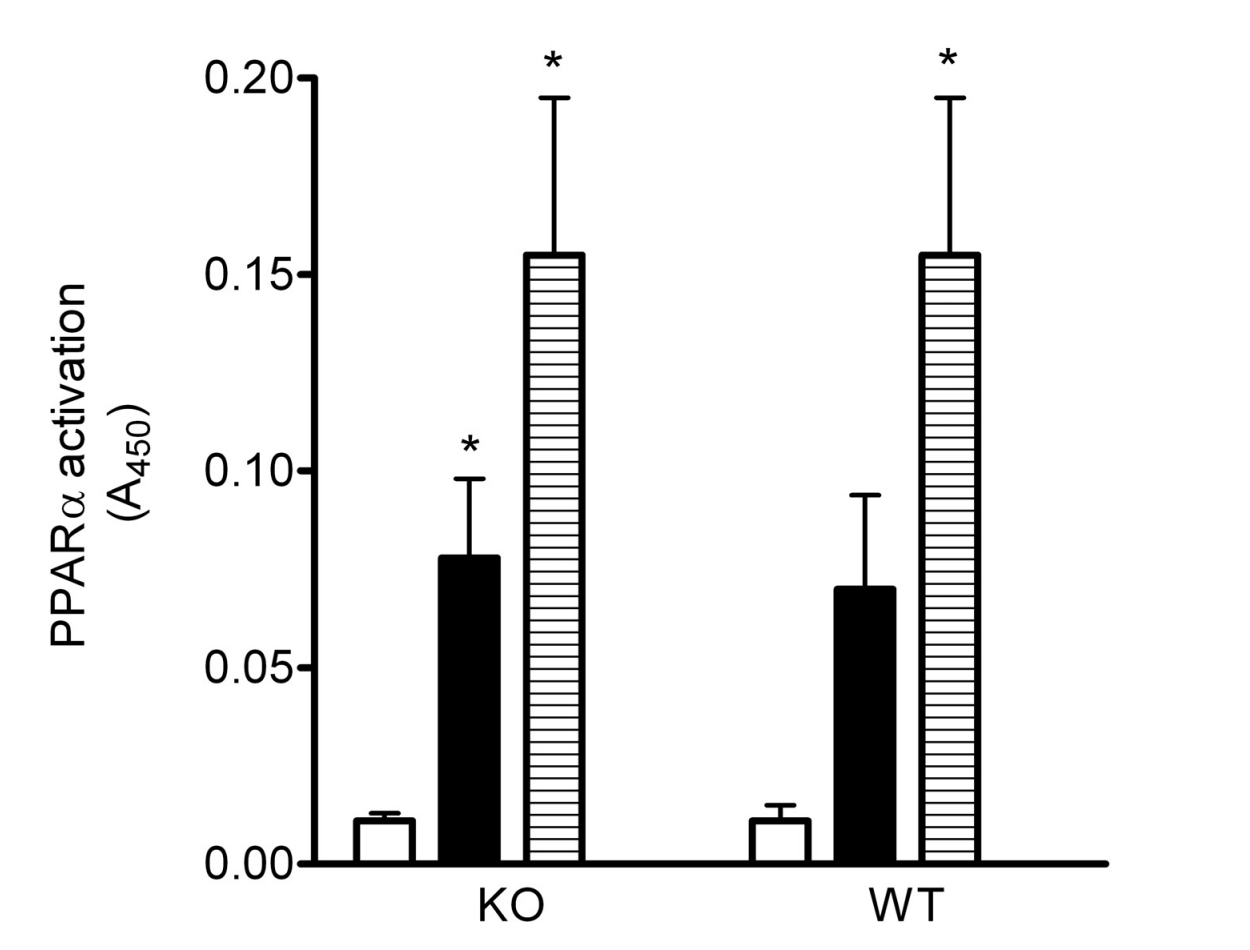
Activation of PPARα by PA and OA in pancreatic islets. KO and WT islets were cultured for 24 with BSA, PA or OA, then nuclear extract was prepared and PPARα activity was measured. Treatment of KO and WT islets with OA significantly increased the PPARα activity (*P < 0.05 vs BSA). The activity of PPARα significantly increased only in KO islets treated with PA (*P < 0.05) compared to BSA. Data are presented as means ± SE, N = 4 or greater for each group.

PPARα activity was determined by a binding assay using nuclear protein from WT and UCP2KO islets and an oligonucleotide corresponding to the PPARα consensus sequence. Treatment of UCP2KO and WT pancreatic islets with OA activated PPARα to a similar extent (~20-fold, Figure 6). Treatment of UCP2 KO and WT islets with PA activated PPARα by ~10-fold but only in UCP2 KO islets was it statistically significant (Figure 6).

### Expression of genes transcriptionally regulated by PPARα

**Figure 7:**
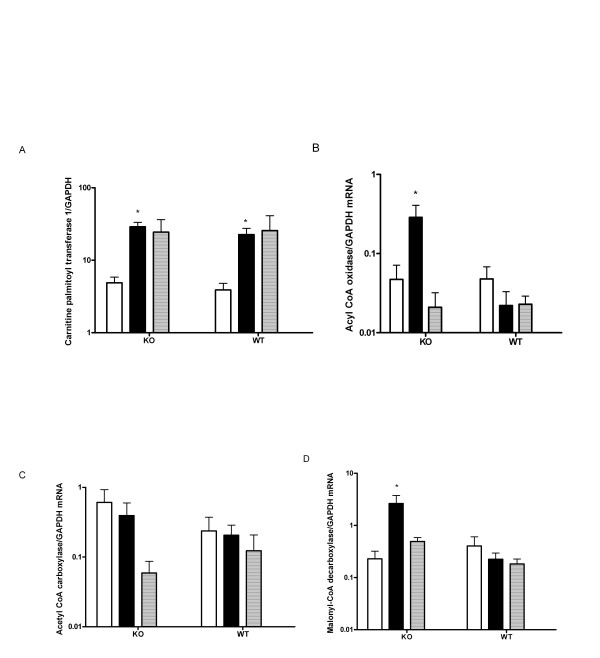
mRNA expression of lipolytic (CPT1, ACO and MCD) and lipogenic (ACC) enzymes were measured by quantitative RT-PCR in KO and WT islets after 24 h incubation with either BSA (open bars), PA (closed bars) or OA (dashed bars). Baseline expression (BSA-cultured islets) was similar in both genotypes for all enzymes. The expression of CPTI was significantly induced (*P < 0.05 vs BSA) in UCP2 KO and WT islets by PA treatment (**7A**). The expression of ACO and MCD was significantly increased only in KO islets treated with PA (**7B & 7D**). In KO and WT islets, the expression of ACC was not affected by PA or OA (**7C**). Data are presented as means ± SE, N = 6–8 for each group.

The expression levels of two classical PPARα target genes, carnitine palmitoyl transferase I (CPTI) and acyl CoA oxidase (ACO) were quantified by real time PCR (Figure 7A,B). CPTI and ACO are rate-limiting enzymes for mitochondrial and peroxisomal fatty acid oxidation, respectively. Baseline expression (in BSA-cultured islets) was similar in both genotypes for all enzymes. The expression of CPTI was significantly induced (~10-fold) in UCP2KO and WT islets by PA treatment (Figure 7A). The induction of CPTI by OA did not achieve statistical significance. Only in UCP2KO islets exposed to PA was the expression of ACO was significantly induced by ~10-fold. In addition, the mRNA of enzymes controlling malonyl CoA concentrations, ACC and MCD was quantified (Figure 7C,D). The expression of MCD was significantly increased in PA-treated islets of KO mice by ~10-fold but no other significant changes were seen.

### Fatty acid oxidation in islets

**Figure 8:**
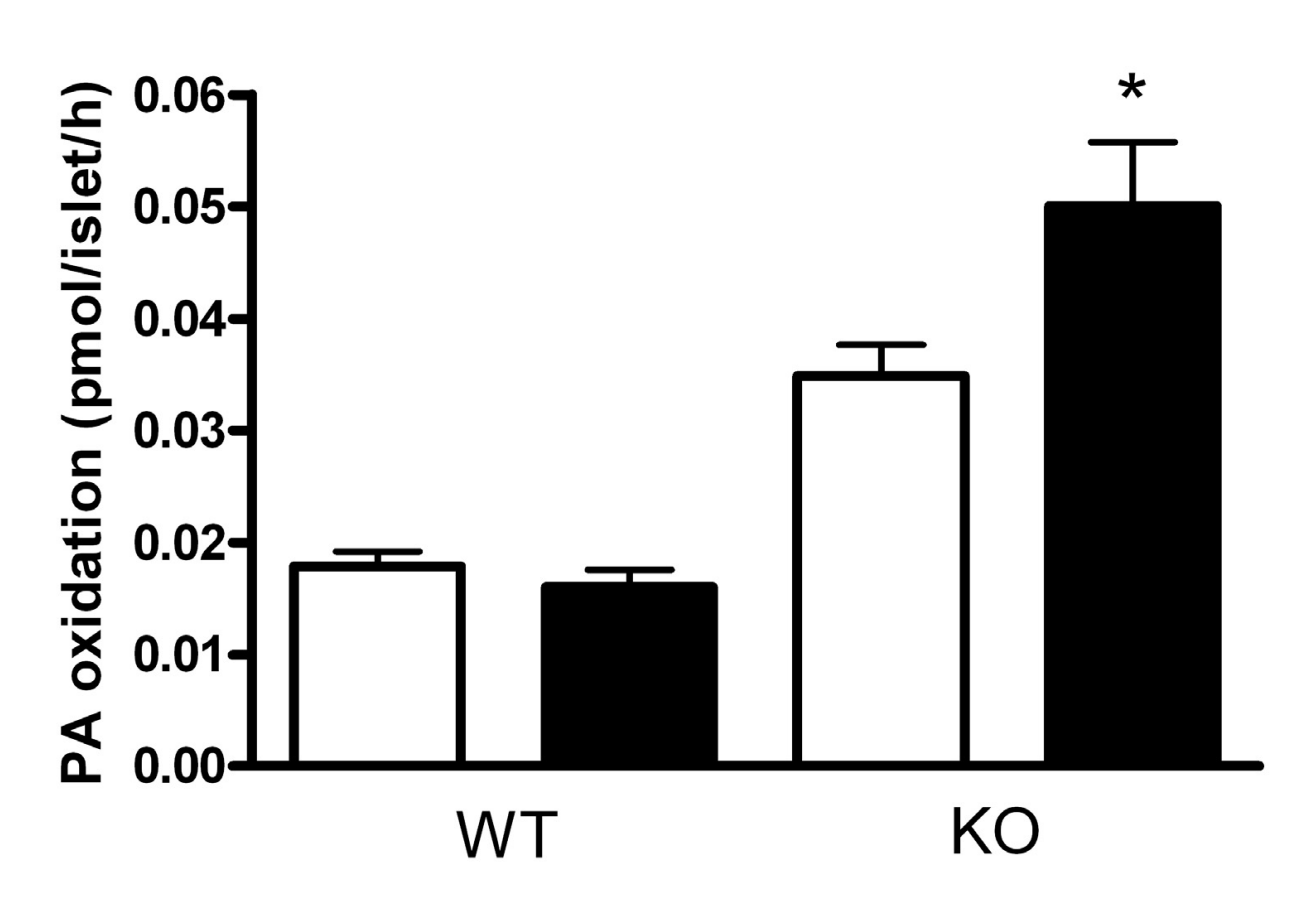
Fatty acid oxidation in islets from WT and UCP2 KO mice. Islets cultured 24 h in control (1% BSA, open bars) or PA (0.4 mM + BSA, closed bars) were incubated with ^14^C-PA and 2.8 mM glucose for 2 h and the evolved ^14^CO_2 _was used to estimate fatty acid oxidation (n = 8 for all). * P < 0.05 comparing UCP2 KO to WT islets.

To assess whether changes in gene expression affected fatty acid oxidation, production of ^14^CO_2 _from ^14^C-PA was measured. In BSA-cultured islets, fatty acid oxidation was ~2-fold higher in islets from UCP2KO compared with WT mice. In WT islets, 24 h exposure to PA had no significant effect on fatty acid oxidation whereas PA increased (P < 0.05) fatty acid oxidation in islets fromUCP2 KO mice (Figure 8).

## Discussion

The present study examined the interaction between PPARα and UCP2 in pancreatic beta cells in situations of low and elevated FFA to test the hypothesis that in UCP2KO mice, protection of beta cells from fatty acid exposure is dependent upon transcriptional events mediated by PPARα. We compared the endogenous expression and activity of PPARα as well as the effects of knocking down PPARα on function of pancreatic islets isolated from UCP2KO and WT mice, in the presence of PA (C16:0) and OA (C18:1). We chose PA and OA because they are the most prevalent saturated and monounsaturated FFA in the circulation. The detrimental effects of saturated FFA on insulin secretion are well known but the underlying mechanisms are still being elucidated. A high fat diet or FFA exposure may cause beta cell lipotoxicity via induction of UCP2 [3,8,9,23]. Notably, null expression of UCP2 protects beta cells from lipotoxicity [8,9], possibly by increasing capacity for fatty acid oxidation [9]. This observation raises the possibility that the transcription factor PPARα, which regulates expression of genes involved in fatty acid oxidation, may modulate the effects of FFA in UCP2KO mouse islets.

Although the physiological role of PPARα in pancreatic islets has been intensively investigated, the results are conflicting. Under normal physiological conditions, beta cell expression of PPARα exceeds that of PPARγ in rodents [24]. However, different stimuli (such as fatty acids and glucose) may change the levels of PPAR subtypes in the pancreatic islets. The main physiological function of PPARα is the regulation of lipid uptake and oxidation, contrasting with that of PPARγ, which promotes uptake but also storage as triacylglycerides [17]. A beneficial effect of PPARα on the function of pancreatic islets is not supported by the study of Tordjman et al [14], who showed that PPARα ectopically expressed in insulinoma cells could induce lipid accumulation along with a significant reduction in GSIS, possibly through induction of UCP2. Conversely, others [17,25] have shown that PPARα has a protective role in islets under conditions of fatty acid treatment or in ob/ob mice [26] and can promote insulin secretion.

To distinguish the acute effects of PPARα on the function of pancreatic islets, we chose the short-term gene suppression by siRNA. In our study, siPPARα potentiated GSIS both in UCP2KO and WT islets cultured under control conditions. This finding supports the data of others showing that PPARα inhibits GSIS [27] but suggests that UCP2 [14] is not the only mechanism through which PPARα exerts its negative effects. This is supported by our data showing that siPPARα did not reduce UCP2 mRNA expression in WT islets. The control conditions in these experiments resulted in exposing the beta cells to very low concentrations of FFA because the DMEM was serum-free and contained 1% defatted BSA. Lowering FFA reduces GSIS [28], possibly by reducing intracellular lipid-derived signaling molecules that stimulate insulin secretion [29]. Reducing PPARα expression would therefore be predicted to enhance GSIS by decreasing fatty acid oxidation and allow accumulation of the stimulatory mediators. However, the effect of siPPARα on basal insulin secretion and GSIS was more pronounced in UCP2KO compared to WT islets. This may be due to increased beta cell ATP at both basal and stimulated glucose concentrations in the UCP2KO mice [30] or it may reflect other unknown beneficial effects of decreasing UCP2 expression. Another recent study showed that beta cell specific overexpression of UCP2 did not decrease GSIS [31]. The reasons for this discrepancy (the effects of UCP2 KO vs UCP2 overexpression on GSIS) are not clear given that several other independent groups have shown regulatory effects of UCP2 on insulin secretion using both over- and null-expression models [32-35].

The expression level of PPARα modified the effect of PA on GSIS in UCP2KO but not WT islets. Whereas in siC islets, PA enhanced insulin secretion from UCP2KO beta cells as noted previously [8,9], the knockdown of PPARα eliminated this effect. This suggests that the potentiating effect of PA on GSIS in UCP2KO mice is mediated by PPARα. Under the same conditions OA did not change insulin secretion in response to 16.5 mM glucose. Others have also noted differences in effects on insulin secretion of saturated versus monounsaturated fatty acids [36]. The lack of effect of PA treatment in WT islets, independent of PPARα status, is further evidence of an interaction between UCP2 and PPARα. Failure to inhibit insulin secretion in WT beta cells occurred in spite of UCP2 mRNA and protein induction by PA in these islets. Also, because siPPARα did not affect UCP2 expression in WT islets basally or with fatty acid exposure, other mechanisms for fatty acid-mediated transcriptional regulation of UCP2 must exist. One possibility is PPARγ[37], which has been implicated previously in induction of UCP2 after fatty acid exposure [3]. Interestingly, hyperlipidemic PPARαKO ob/ob mice had impaired GSIS and low expression of enzymes involved in fatty acid oxidation [26]. Although moderate levels of esterified intracellular lipids may benefit GSIS, impairment of fatty acid oxidation can lead to increased ceramide formation [36] and/or activation of PPARγ (to compensate for the lack of PPARα, [38]). Whatever the case, the expression level of PPARα appears to determine whether PA potentiates or impairs GSIS in UCP2 KO islets. It is also important to mention that UCP2 may increase fatty acid cycling; therefore, an increase in UCP2 does not necessarily translate into an increase in fatty acid oxidation if it exports FA from the mitochondrial matrix before they can be oxidized, as has been proposed for UCP3 [39,40].

The altered effects of PPARα in UCP2 KO mice might be caused by either a change in expression levels or a change in activity of the transcription factor. However, absence of UCP2 did not modify baseline PPARα protein expression or basal nuclear translocation compared to WT islets. PA and OA increased both PPARα expression and transactivation in UCP2 KO islets but had a significant effect only on transactivation in WT islets, such that total activation was similar between the genotypes. Monounsaturated fatty acids are known to be more effective ligands of PPARα than saturated fatty acids [41] and this was true in this study also. The similar activation levels of PPARα by PA in UCP2KO and WT islets, yet a clear PPARα-dependence of PA to exert beneficial effects on GSIS in UCP2 seems paradoxical. One explanation is that the sensitivity to PPARα of target genes is enhanced in the absence of UCP2 by an as yet undefined effect on co-activators or -repressors of PPARα. Moreover, interaction of PPARα co-activators is ligand-dependent [42], which might explain differential effects of PA and OA on gene transcription, as we describe below.

Enhanced fatty acid oxidation has been speculated to limit formation of toxic lipid byproducts during chronic exposure to saturated fatty acids in UCP2KO mouse islets [9]. In this study, PA oxidation was enhanced in control islets from UCP2KO mice and increased further after culture of islets in PA for 24 h. Carnitine palmitoyltransferase I (CPTI) catalyzes the transfer of fatty acids from CoA to carnitine, allowing the initial transport of fatty acids into mitochondria for β-oxidation. Its activity and expression are highly regulated and rate limiting. CPTI gene expression is regulated by PPARα [43]. Although gene expression levels do not necessarily equate to functional activity it is of interest to point out that UCP2KO and WT islets treated with PA had similarly elevated CPTI mRNA expression. Unless there are differences in translation between the two genotypes, then CPTI expression cannot account for increased fatty acid oxidation in these experiments. This is in contrast to the result of [9], but FFA exposure was different in that study (4.5 months) compared to the present study (24 h).

Malonyl-CoA is a metabolic signaling molecule that regulates lipid partitioning through its inhibitory action on CPTI. Malonyl-CoA levels change in different physiological and pathological conditions [44]. For example, in ischemia- reperfusion, which is associated with a significant increase in fatty acid oxidation, malonyl-CoA levels decrease rapidly [45]. Inhibition of CPTI by malonyl-CoA leads to a decrease in the uptake of fatty acids into the mitochondria, which results in decreasing mitochondrial fatty acid oxidation [46]. Malonyl-CoA concentrations can be regulated through synthesis, degradation or both. Acetyl CoA carboxylase (ACC) is the rate-limiting enzyme in the synthesis and regulation of malonyl-CoA. In our study the ACC expression was not different among UCP2KO or WT islets. In contrast, we found that malonyl-CoA decarboxylase (MCD) expression in UCP2 KO islets was induced by 24 h PA treatment, which would be predicted to reduce malonyl-CoA levels and enhance fatty acid oxidation, as has been found in muscle [47]. Also, conditions that increase hepatic fatty acid oxidation (such as streptozotocin-induced diabetes or a 48 h fast) increase MCD activity [48]. In disease states such as hyperlipidemia and diabetes, the important role of MCD in the regulation of fatty acid metabolism, through altering the cytoplasmic levels of malonyl-CoA, is known [49].

Finally, fatty acid oxidation outside of the mitochondrion may also be increased to account for the changes seen in UCP2KO mouse islets. Acyl CoA oxidase (ACO) catalyzes the first step in peroxisomal fatty acid oxidation [50]. Although the basal expression of ACO was similar in UCP2KO vs WT islets, only in the absence of UCP2 did PA induce this enzyme, suggesting that peroxisomal fatty acid oxidation may also contribute to enhanced fatty acid oxidation in UCP2KO islets.

## Conclusion

In the absence of UCP2, the effects of PPARα activation on transcription of fatty acid oxidation-promoting enzymes (ACO, CPT-I and MCD) precipitate an intracellular environment favouring fatty acid oxidation to reduce lipotoxicity and enhance GSIS in the presence of saturated fatty acid. In the short term, this effect appears to be mediated mainly by differential induction of ACO and MCD, whereas in the longer term CPTI may also be increased in UCP2KO relative to WT islets [8]. Conversely, knockdown of PPARα in UCP2KO islets leaves them vulnerable to detrimental effects of saturated fatty acids. This could occur if MCD and ACO were not up-regulated, reducing fatty acid oxidative capacity. Notably, MCD expression is elevated in heart of PPARα KO mice, concomitant with impaired fatty acid oxidation [51]. This hypothesis could be examined in islets in future. In fact very recently, the effectiveness of MCD inhibitors on ischemic heart disease have been reported [52]. Moreover, because PPARα expression and activation were similar in UCP2KO and WT islets, and because OA induced PPARα but did not promote GSIS, we suggest that other molecules, yet to be identified, are also involved in regulating the differential response of UCP2KO islets to PA.

## Abbreviations

PPARα: peroxisome proliferator-activated receptor-α, UCP2: uncoupling protein-2, PA: Palmitic acid, OA: Oleic acid, GSIS: glucose stimulated insulin secretion, FFA: Free fatty acids, TTBS: Tween Tris-buffered saline, GAPDH: Glyceraldehyde-3-phosphate dehyrogenase, CPTI: carnitine palmitoyltransferase I, ACO: acyl CoA oxidase, ACC: Acetyl CoA carboxylase, MCD: malonyl CoA decarboxylase, EGFP: enhanced green fluorescence protein, KO: knockout, WT: wild-type, DMEM: Dulbecco's modified Eagle medium.

## Competing interests

The author(s) declare that they have no competing interests.
